# Outcomes of a risk assessment and management program using telecare consultation among patients with diabetes mellitus in general out-patient clinic: a hybrid effectiveness-implementation study protocol

**DOI:** 10.1080/07853890.2023.2262088

**Published:** 2023-09-25

**Authors:** Arkers Kwan Ching Wong, Frances Kam Yuet Wong, Jun Liang, Danny Wah Kun Tong, Man Li Chan, Tsun kit Chu, Bo Chu Wong, Rinis Sin Yi Chan, Wai Hing Ho, Cecilia Yeuk Sze Tang, Sau Ching Chiang

**Affiliations:** a School of Nursing, The Hong Kong Polytechnic University, Hung Hom, Hong Kong; b New Territories West Cluster, Hospital Authority, Homantin, Hong Kong

**Keywords:** Diabetes, telecare, implementation science, hybrid effectiveness-implementation, clinic, consultation

## Abstract

Diabetes Mellitus (DM) is a chronic disease characterized by abnormally uncontrolled high blood glucose level. The Risk Assessment and Management Program (RAMP) in Hong Kong has been providing long-term face-to-face follow-up to DM patients in the government out-patient clinics since 2009. However, under the current outbreak of COVID-19, these face-to-face consultations were ceased over and over again to lower the risk of disease transmission. With the advancement in technology, the recent emergence of telecare has provided an alternative to replace the conventional consultations in the clinics. Its clinical effectiveness on DM patients has also been supported by numerous studies. Yet, there is only a paucity of literatures discussing the practicality of such implementation design in the real-world settings. This study aims at studying both the effectiveness and implementation outcomes of telecare in Hong Kong DM patients. It adopts a type 2 hybrid effectiveness-implementation design. It will be conducted in seven government out-patient clinics in Hong Kong. The subjects will be randomly assigned to an intervention group or a control group when they 1) are aged 18 or above, 2) have a confirmed diagnosis of diabetes, and 3) are having regular follow-up appointment in the clinic. Subjects in the intervention group will receive a 84-week Risk Assessment and Management Program (RAMP) in an alternate telecare and face-to-face consultations mode, while the control group will receive the same program but in usual face-to-face consultation mode. RE-AIM is employed as the implementation and effectiveness outcome evaluation framework. The primary outcome measure will be HbA1c. Data will be collected pre-intervention (T1), 42-week (T2), and 84-week (T3). The study will provide effectiveness-implementation assessment of telecare mode for DM patients in Hong Kong, as an alternative or in addition to conventional face-to-face consultations. It also aimed to provide insights for the future adoption in a broader health care setting.

## Introduction

Diabetes Mellitus (DM) is a chronic, metabolic disease characterized by abnormally uncontrolled high blood glucose levels [1]. Its prevalence has risen dramatically over the past decades, reaching approximately 529 million people worldwide in 2021. It is even estimated that the figures will be doubled to 1.31 billion by 2050 [2]. In Hong Kong, 10% of the total population is affected by DM and is expected to rise to 12.8% by 2025 [3]. According to a recent study, the incidence of DM increased gradually in people aged 20 to 40 [4], implying that the DM population in Hong Kong not only becomes larger, but younger. DM can lead to a wide range of microvascular and macrovascular impacts. It is reported that 40% of patients have more than one complication such as angiopathy, retinopathy, cardiovascular diseases, and cerebrovascular diseases [5]. In addition, DM is posing a heavy burden to the healthcare systems and economy as the treatment of DM requires extensive use of medical resources. According to a study in Hong Kong, it mentioned that approximately $2 billion were spent each year as the health care cost for DM [6]. Furthermore, patients with severe complications might not be able to work. This could result in a decline in the working population, which is unfavourable to the society in the long run.

DM isn’t a disease that can be fully cured but requires a long-term follow-up and management in the community. In Hong Kong, the General Out-Patient Clinics (GOPCs) under the Hospital Authority have served millions of patients with chronic illness, including DM [7]. Since 2009, there is a program named the Risk Assessment Management Program (RAMP) that initiates in GOPCs intending to provide multidisciplinary risk assessment, complication screenings, and subsequent regular follow-up consultations to DM patients (Appendix 1 showed the RAMP workflow). Throughout these years, researchers have already proven the significant clinical effectiveness of the RAMP in terms of patients’ health outcomes such as improvement in HbA1c, blood pressure, lipid profile, and BMI [8–10]. Yet, these conventional face-to-face consultations could be time-consuming and troublesome owing to geographical issues and traveling difficulties. To illustrate, some patients might be living in far-off areas which could take them quite a lot of time to arrive at the clinic. More importantly, hospital visits greatly increase the risk of disease transmission, especially under the current COVID-19 pandemic. With the advance of science and technology, telecare consultation could be a flexible and cost-effective alternative method to continue thus consummate our support to these DM patients.

Telecare consultation is a type of virtual consultation where communication takes place between healthcare professionals and clients through telecommunication applications. In a previous study utilizing a survey design, it was found that the majority of patients who needed to contact general practices during the COVID-19 lockdown period were highly satisfied with telecare for routine consultations in terms of convenience and safety [11]. When focuses on the clinical effectiveness of telecare on DM care, a past study acknowledged that such mode has a positive impact on health indicators such as blood glucose level and lipid [12]. Moreover, a previous 5-year longitudinal study showed that using telecare as a consultation mode has led to better HbA1c, blood pressure levels, and lipid profile among DM patients than adopting the conventional face-to-face consultations [13].

While telecare consultation has its advantages over conventional consultation in managing the health outcomes of DM patients as supported by a great number of literatures, the practicality and achievability of telecare in real-world settings have yet to be explored. Possible barriers such as insufficient digital literacy, lack of access to technology, and absence of clear operational guidelines may limit the willingness of healthcare professionals to implement the intervention [14]. More importantly, economic considerations like whether the intervention meets economic principles, are all key determinants of the practicability of applying and sustaining telecare consultation in healthcare organizations. Several overseas studies with an implementation science basis have explored perceived conditions to ensure the successful implementation of telecare. For example, the majority of respondents agreed that the easiness of telecare use and its compatibility with daily life are key factors that may influence their use of telecare consultation. From a socio-economical aspect, respondents commented that it is important to have an adequate amount of financial aid to support their sustained adoption of telecare. Additionally, a safe telecare network is also crucial to protect data confidentiality as well as patient privacy [15, 16].

Implementation science provides insight for integrating new programs, such as telecare, into the healthcare system by evaluating the clinical effectiveness as well as the practicality in real-world settings. Currently, the use of telecare consultation is limited despite an outstanding telecommunication infrastructure in Hong Kong [17]. The ongoing COVID-19 pandemic creates a pressing need for telecare consultation to be integrated into the current RAMP in hopes to continue the care for DM patients. To the best of our knowledge, there are no translational researches available that explore simultaneously the effectiveness and implementation outcomes of adopting telecare model of care in a primary health care setting in Hong Kong. If this proves successful, this would be the first study locally to provide insightful understandings on the execution and maintenance of telecare consultations in not only DM care, but also in other chronic disease management in primary care settings.

## Conceptual framework

The hybrid effectiveness-implementation design of this study is guided by two models. The design of the implementation strategies is informed by the Generic Implementation Framework (GIF) [18], while the design of the effectiveness and implementation evaluations is based on the RE-AIM framework [19].

The GIF proposes that there are facilitating and hindering factors, and strategies influencing each stage of implementation process, namely, development and exploration, preparation, operation, sustainability phases [18]. Facilitating factors such as support from management level and hindering factors such as low staff engagement level may support or hamper the dissemination and implementation process of the program. The present study will therefore utilise the GIF as a foundation, and address the facilitating and hindering factors by providing appropriate implementation strategies, such as staff training and program orientation, in different stages of the implementation process.

RE-AIM is an evaluation framework to guide the effectiveness and implementation outcomes evaluation [19]. RE-AIM examines 5 dimensions: *Reach* into the target population, *Effectiveness* of the intervention, *Adoption* by the staff and setting, *Implementation* fidelity, and *Maintenance* (i.e. degree of sustainment over time). Thus, RE-AIM is ideal for structuring the proposed hybrid implementation-effectiveness evaluation. The results of these evaluations are expected to lead to more profound insights in the feasibility and translatability of the intervention, optimal training for the staff and patients, and the factors influencing the acceptance of the intervention by the target population.

## Aims

There are two aims for the present study: 1) compare the clinical effectiveness between alternate telecare and face-to-face consultations and usual face-to-face consultations in improving client outcomes (i.e. HbA1c, Body Mass Index [BMI], fasting lipid profile, medication adherence, quality of life [QoL]) and health service utilization (i.e. unscheduled attendance at general practitioners’ office, emergency department, general out-patient clinic) among DM patients, and 2) explore and identify whether an implementation strategy of alternate telecare and face-to-face consultations at general out-patient clinics is reachable to the target DM patients, adoptable to the clinic staff and clinic, implementing accurately, and sustainable in long term.

## Hypothesis

It is hypothesized that DM patients who receive alternate telecare and face-to-face consultations will have no differences in client outcomes and health service utilization outcomes compared to those who receive usual face-to-face consultations.

## Methods/design

### Study design

Hybrid effectiveness-implementation type 2 design is adopted in this study. It is a design that evaluates the effectiveness of an intervention while also considers the implementation process and factors that may influence its successful adoption and integration into real-world settings [20]. Type2 design is used when intervention in both the clinical and implementation spheres are tested simultaneously. In this study, a randomized controlled trial will be used to compare the effectiveness between alternate telecare and face-to-face consultations and usual face-to-face consultations, while both qualitative and quantitative methods will be employed to evaluate implementation strategies and determine the factors that may influence implementation process.

### Study setting, subjects and recruitment strategies

The study will be implemented in seven government out-patient clinics (GOPCs) under Hospital Authority in the New Territories West Cluster from Jan 2023 to Dec 2025. The subjects who are DM patients (either type 1 or type 2) with regular follow-up appointment in the clinic will be screened and recruited into the study if they 1) are aged 18 or above, and 2) have a confirmed diagnosis of diabetes. They will be excluded if they are 1) having dementia, 2) having unaccompanied hearing or visual loss, 3) not living in an area with Internet coverage.

Research assistants (RAs) will introduce the program to the eligible DM patients in the clinics. For those who are eligible and agreed to join, RAs will collect their signature for the consent form and baseline data. After successfully collect their data, the RAs will call the principal investigator (PI) of the research team for randomization. The PI will put the numbers (i.e. 1 = “telecare group”, 2 = “control group”) that generated from a software “Research Randomizer” into sealed envelopes and open sequentially when receive the call from RAs. A block randomization of size 8 will be employed to ensure balanced representation of the subjects in each group. The PI will provide the group list to the clinic clerk, who will then inform individual participants about their randomization results and guide them to follow the assigned delivery consultation mode. The Research Assistants (RAs), who will solely be involved in data collection, and the statistician, who will be responsible for data analysis, will be blinded to the group assignment.

### Implementation process

#### Development and exploration phase

The research team has met with the chief of service, practitioners, and the managers of the GOPCs for three times to introduce the program, discuss about the impacts of the program to the GOPCs and the DM patients, and the pros and cons. With the support of the chief of service and the staff, both parties further discuss about the strategies and logistics of adopting the telecare consultation in GOPCs. The research team has agreed to a) promote the program within the GOPCs by preparing pamphlets and posters; and b) support the program’s implementation and adoption process.

#### Preparation phase

Before commencement of the program, program orientation and training workshops have to be provided to the staff in GOPCs. The research team has introduced and explained the program, illustrated the logistics of the program, and the roles and responsibilities of each healthcare providers in the one-day workshop. Each participant also received a user guide that displays the program workflow and explains with easy text and graphic instructions how to use the telecare tool. In addition, recruitment and implementation process protocols were developed with the collaboration of the healthcare providers in the GOPCs. The Information Technology (IT) team of the Cluster will provide IT support during the whole process.

#### Operation phase

Both intervention (alternate telecare and face-to-face consultation) and control (usual face-to-face consultation) group subjects will receive DM RAMP in one of the seven GOPCs. Patients enrolled in the RAMP will undergo an initial risk assessment, which include physical examination, eye and foot assessment, laboratory testing, and complication screening. According to their conditions, the healthcare providers, including nurse, practitioners, and physiotherapists, will provide individualized diabetes education and lifestyle modification advice to the DM patients [21]. In this study, the healthcare providers for both groups will be the same to minimize the operator effect. The only difference in these two groups is the delivery model, where intervention group will receive alternate telecare and face-to-face consultations and control group will receive usual face-to-face consultations only within the 84-week study period. During this program duration, there will be a total of seven consultations for both groups as each consultation is approximately 14 weeks apart. Both groups will receive the first consultation onsite. Before the end of the first consultation, the healthcare providers will provide intervention group patients instructions on how to use HA Go, a telecommunication app that can provide virtual face-to-face service, in their next meeting. A clerk will help contact the patients one hour prior to the next consultation for rehearsal. Any technical problems will be managed in this one hour by either the clerk or the IT team.

#### Sustainability phase

The research team and the healthcare providers will discuss the implementation progress at bimonthly meetings, and if necessary, adjustment will be made. The changes will be documented throughout the process. Since the implementation of telecare consultation is still in its infancy phase for the Hospital Authority and its use is increasing, particularly during the COVID-19 pandemic, the corporate policy and guidelines of the service may be modified during the operation phase of this study.

### Sample size

Power analysis is used to estimate the sample size of the study. Based on the non-inferiority criteria of 0.4% between the two groups on the primary outcome, HbA1c, in a previous similar study [22], and the varying drop-out rates of previous studies, which ranged from 0% to 20% over a follow-up period of 1–10 years [23–26], assuming a 20% of beta error, a 0.05 of alpha level, and a drop-out rate of 20% in this study, a sample size of 350 subjects will be needed in each group, i.e. a total of 700 subjects in two groups.

### Ethics and dissemination

Ethical approval was granted from the ethics subcommittee of a university in Hong Kong. Information of significance, aims, procedures, benefits and risks will be told to all eligible subjects. Subjects will be reassured that they can refuse participation and withdraw from the study anytime without any penalty. Subjects will sign the consent form after they expressed understanding of the study. They will remain anonymous, and all data will be identified by a case number only. A telephone hotline will also be provided to the subjects for queries.

### Outcome measures

The present program will use RE-AIM as the evaluation framework.

#### Reach:

To identify how representative the enrolled patients are of the target population, the number of DM patients who are eligible, invited, excluded, and enrolled to receive telecare consultation in the GOPCs will be counted and compared to non-participants on key demographic data such as age, diabetes type and duration, and number of follow-up attendance. The RAs will also try to ask both participating and non-participating patients the reasons why they enrol or not enrol to provide further information on the reach of the program.

#### Effectiveness:

The effectiveness outcomes of the program include HbA1c, fasting lipid profile, body mass index (BMI), medication adherence, quality of life (QoL), and health service utilization. The primary effectiveness outcome of this study is the HbA1c level. HbA1c is a standard assessment of glycemia and refers to the mean blood glucose levels over the previous 2 to 3 months [27]. Fasting lipid profile includes cholesterol level, and high- and low-density lipoprotein. Similar to HbA1c, the fasting lipid profile will be drawn from patients’ blood when the patient has onsite consultation. The results can be extracted from the hospital database. BMI will be measured each time during GOPC admission by dividing weight in kilograms by height in meters^2^. Medication adherence will be measured by adherence to refills and medication scale [28]. This 4-point Likert scale has scores range from 12 to 48, with lower scores representing better medication adherence. The scale has demonstrated high internal consistency, with a Cronbach’s alpha of 0.81 [28]. Quality of life will be evaluated by the version 2 of the 12-item Short Form Health Survey [29]. The scale measures various aspects of physical and mental health from which physical and mental component scores can be summed. It has been widely used in Chinese diabetic patients with good validity and reliability [30,31]. The outcomes of health services utilisation include the total number of unscheduled visits to general out-patient departments, general practitioners (GP), emergency departments, and hospitals, and the total health service attendance. The information will be extracted from the hospital database, except for GP visits, which will be reported by the subjects.

Background demographic data will be collected to compare the baseline differences between the two groups. They include age, gender, marital status, education, work status, years of using a smartphone, financial status, family living in the same household, caretaking support, diabetes type and duration, date of first follow-up, number of referrals, professionals to whom the patient is referred.

#### Adoption:

How success the telecare consultation adopted at the staff and clinic level will be determined by the Readiness for Implementation Model Survey [32]. The survey comprises 42 items asking the healthcare providers about the facilitators and barriers of adopting telecare in GOPC. The scores range from 0 to 100, with higher scores indicating better adoption. The scale has demonstrated good inter-rater reliability [32].

In addition to quantitative survey, separated semi-structured group interviews with healthcare providers and patients will be conducted to explore their views on facilitating, hindering factors, and the feasibility of implementing the program in GOPC.

#### Implementation:

A performance checklist that developed by the PI according to the workflow of the program will be used to identify whether the program is implemented as intended. The checklist was validated by a group of healthcare professionals such as doctors, optometrists, and nurses, and global researchers who have experience in conducting implementation science studies. In addition, five percent of the patient documentation will be extracted from the database of the hospital to check the adherence of the interventions by the healthcare providers to the set protocol.

#### Maintenance:

The sustainability effect of the program will be evaluated by measuring the effectiveness outcomes at 84 weeks after the commencement of the program. At each data collection timepoint (i.e. baseline, 42 weeks, 84 weeks), the RAs will record the number of patients who withdraw from the program or request to change from alternate telecare and face-to-face consultation to usual face-to-face consultation or vice versa and the reasons for doing so. In addition, maintenance of the program will depend on the costing evaluations. The cost of telecare consultation, including the software and on-going costs, will be estimated from the provider perspective as well as from a societal perspective, including patients’ costs in terms of travelling and time. An ingredient approach will be employed to estimate the cost. The total estimated cost for both groups will be estimated and compared.

### Data collection

Data will be collected at three time-intervals, baseline pre-intervention (T1), 42 weeks (T2), and 84 weeks after the interventions begin (T3). The RAs, who are blinded to group allocation and not involved in interventions, will collect the data in the GOPCs in T1, and *via* telephone call in T2 and T3. They will be tested on inter-rater reliability to ensure data quality. Five percent of the data will also be randomly selected for independent review. Participants will receive a HK$ 50 coupon as a token of appreciation for their time commitment and to help cover transportation costs upon completion of the T3 data collection.

Semi-structured interviews will be carried out in T3 by research team members. A one-time in-depth focus group interview allows the research team to understand the group dynamics where different opinions from persons can be explored. During the interview, participants will be asked questions such as "What is your experience with the telecare consultation?" and "What aspects of the intervention program do you like or dislike?" At each time point, separated interviews will be conducted to the same group of people including (1) GOPC manager, (2) clinic healthcare providers (i.e. doctor, nurse), and (3) 64 (20%) of the patients in telecare group.

### Data analysis

The Statistical Package for Social Sciences version 26 software will be used to analyse the quantitative data. The independent samples t-test will be used to calculate the difference between the alternate telecare and face-to-face consultation usual face-to-face consultation groups, and the null hypothesis will be rejected if the upper bound of the 95% 1-sided confidence interval is smaller than the non-inferiority margin, which sets at 0.4%. Regression using two-way interactions between the study group (telecare versus conventional) and a range of demographic data such as age, gender, and diabetes type and duration will be performed to examine the subgroup effects.

The principles of thematic analysis will be used to analyse the qualitative data gathered in the semi-structured interviews. All focus group interviews will be audio-recorded and transcribed. The research team members will independently examine the raw text and identify relevant themes. The team will discuss and construct a framework for analysis with clearly defined codes and categories, which will help in summarizing the data in order to answer the research questions. An audit trail will be kept to ensure consistency in the coding and interpretation, and all discrepancies will be resolved by consensus.

## Discussion

The purpose of this study is to investigate both the implementation and effectiveness outcomes of a telecare consultation program on long-term care services for DM patients in Hong Kong. Although there has been an adequate amount of research looking into the clinical effectiveness of telecare on DM care, these studies have shown weaknesses in considering the barriers and obstacles in implementing telecare in a real-world setting. Aiming to bridge this very gap, our proposed study may be insightful and inspiring for future adaptation of telecare in diverse chronic disease managements in Hong Kong primary care settings.

Throughout the program, the intervention group will be attending a total of 7 alternate virtual and face-to-face consultations within 84 weeks, while the control group will be having complete conventional face-to-face consultations. Clinical outcomes such as BMI, blood glucose level, fasting lipid profile, and most importantly HbA1c will be measured and compared at three distinct time points. Simultaneously, implementation strategies of telecare consultation will be evaluated in the aspects of the reach into the target population, adoption by the staff and setting, implementation fidelity, and maintenance as guided by the RE-AIM framework. To the best of our knowledge, this study is the pioneering attempt in Hong Kong to adopt a hybrid design to examine outcomes and effects. The strength of this study include the fact that the program has been receiving unlimited supported by the key stakeholders, including the managerial level and frontline staff of general out-patient clinics. The implementation and logistic protocols, training workshops, and the provider guidelines were co-designed by the research and the service teams. The proposed telecare consultation model is thus more likely to be sustained in reality, since it is built with the engagement of the service partner.

The study findings can provide evidence to support policy makers and healthcare providers in service design and delivery. If proven effective, this program has important implications for patients who are isolated by either their physical disabilities, distant living location or the pandemic situation, which may be recurrent. In addition, given that most of the DM patients are full-time workers, to prevent the loss of work and salary being cut, report showed that they have the tendency to skip the medical appointment occasionally [33,34]. Non-attendance not only has an impact on patients’ health outcomes, but also influence the capacity and financial stability of clinics. The implementation and sustainment of the telecare service to these employees may help save their traveling and waiting time in the clinic and prevent the issues of non-attendance of healthcare appointments. .

The present study may also have an impact on the healthcare system by opening up new avenues of care delivery and laying the groundwork for the integration of this technological innovation into routine care services. Once the facilitators and barriers that may support or hinder the dissemination and implementation of the telecare program into the primary care setting were identified, the management level staff will be able to apply strategies to assist the implementation process. The healthcare professionals will also help reach a better triage by identifying the clinically stable and suitable patients to join the telecare services, which immediately reduce the waiting times for those who are in urgency for medical contact and minimize the overcrowding of the clinic. The IT team of the hospital will get more familiarize with the installation, implementation, and troubleshooting of the HA Go and the telecare services, and eventually provide speedy and quality technical support to the patients.

Although the strengths of the program have been identified, we also anticipate difficulties during implementation. Firstly, some of our clients might not be able to access to the internet which would impede the implementation of the online telecare consultation. For example, elderlies could have troubles setting up internet appliance such as Wi-Fi [35], while some might not be able to afford any of this equipment. In light of this situation, our team would be providing these clients with Internet and the corresponding handling instructions so that they can use it at home to access the HA Go. Secondly, considering the possible technical errors that may happen during the telecare consultations, we would have an IT team in GOPCs to help fix the problem immediately, hoping the consultations can be gone through smoothly.

## Conclusion

While the global government has, in the light of the outbreak of the Coronavirus Disease 2019 epidemic, supported the implementation of telecare services to improve the reach, satisfaction, and health conditions of the patients with chronic diseases, there is still no translational research simultaneously implementing and evaluating this telecare model of care delivered in a primary care setting. The present study may provide management level of healthcare service and policy makers key insights into the mechanisms and processes for implementing and sustaining telecare consultation in not only the primary care setting, but secondary and tertiary settings, as well as expanding the service scope to cover patients of new cases.

## Data Availability

The datasets used and/or analysed during the current study are available from the corresponding author on reasonable request.
